# Guide to Plant-PET Imaging Using ^11^CO_2_

**DOI:** 10.3389/fpls.2021.602550

**Published:** 2021-06-02

**Authors:** Jens Mincke, Jan Courtyn, Christian Vanhove, Stefaan Vandenberghe, Kathy Steppe

**Affiliations:** ^1^Laboratory of Plant Ecology, Department of Plants and Crops, Faculty of Bioscience Engineering, Ghent University, Ghent, Belgium; ^2^MEDISIP - INFINITY - IBiTech, Department of Electronics and Information Systems, Faculty of Engineering and Architecture, Ghent University, Ghent, Belgium; ^3^Medical Molecular Imaging and Therapy, Department of Radiology and Nuclear Medicine, Ghent University Hospital, Ghent, Belgium

**Keywords:** ^11^CO_2_, carbon-11 (^11^C), positron emission tomography (PET), plant-PET, guide, image analysis, image quantification, positron autoradiography

## Abstract

Due to its high sensitivity and specificity for tumor detection, positron emission tomography (PET) has become a standard and widely used molecular imaging technique. Given the popularity of PET, both clinically and preclinically, its use has been extended to study plants. However, only a limited number of research groups worldwide report PET-based studies, while we believe that this technique has much more potential and could contribute extensively to plant science. The limited application of PET may be related to the complexity of putting together methodological developments from multiple disciplines, such as radio-pharmacology, physics, mathematics and engineering, which may form an obstacle for some research groups. By means of this manuscript, we want to encourage researchers to study plants using PET. The main goal is to provide a clear description on how to design and execute PET scans, process the resulting data and fully explore its potential by quantification via compartmental modeling. The different steps that need to be taken will be discussed as well as the related challenges. Hereby, the main focus will be on, although not limited to, tracing ^11^CO_2_ to study plant carbon dynamics.

## Introduction

**FIGURE 1:**
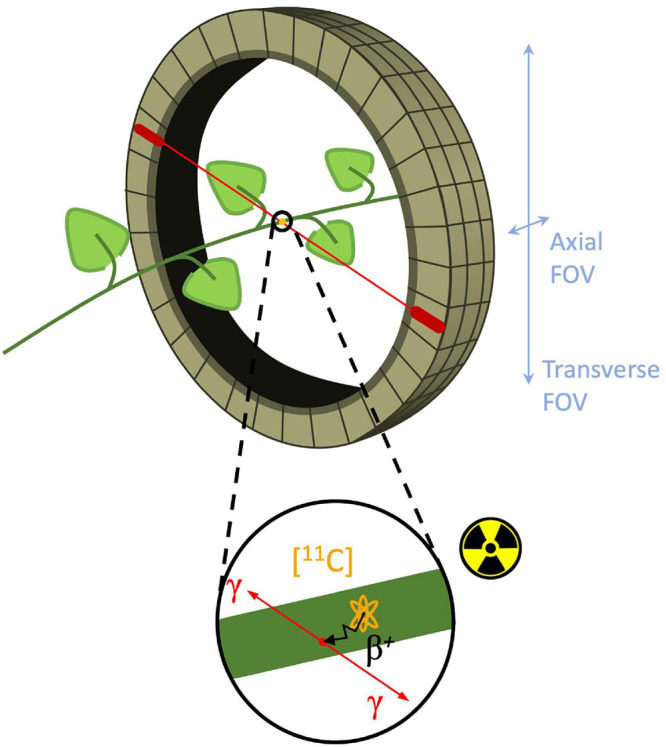
Schematic of branch inside a PET detector ring, i.e., field of view (FOV). Positron decay of the (orange) ^11^C-nucleus in the branch is shown in the enlarged circle. The positron is traveling a certain distance (typically 1.2 mm for ^11^C-positrons - black arrow) known as the positron range to eventually collide with an electron and annihilate to produce two γ-photons (red arrows) traveling in opposite (180°) direction. Subsequently, these γ-photons can be detected by two different PET detectors (red ovals) in the detector ring.

Molecular imaging is a type of medical imaging that has the ability to trace or identify specific molecules within a specific anatomic location and can provide insight into metabolic pathways, tissue components, and tracing solute transport mechanisms (Wickline and Lanza, 2002; James and Gambhir, 2012). Today, molecular imaging is an established tool in both a clinical setting as well as in research facilities, where it is either used for diagnostic imaging and treatment, or for clinical research and drug development. Fueled by the advances and developments of new radioactive labeled probes, functional imaging techniques such as positron emission tomography (PET) and single photon emission computed tomography (SPECT), in combination with computed tomography (CT) or magnetic resonance imaging (MRI) have become increasingly important (Levin, 2005). As in other functional imaging techniques, PET measures *in vivo* distribution and concentration of radiotracers in a non-invasive manner. Radiotracers are molecules that contain two moieties (or functional groups), i.e., an agent that has a high affinity for a specific target that needs the be imaged and a positron emitting label (e.g., ^11^C, ^18^F, and ^15^O) in case of PET (Kiser et al., 2008; Saha, 2016). The emitted positron β^+^ (antimatter of an electron) will react with an electron in its close environment and annihilate. The mass of both particles is hereby converted into energy manifesting as two gamma (γ) photons, which are emitted in opposite direction (180°) to be detected with a ring of detectors (Figure 1; Ametamey et al., 2008; Kim et al., 2013). When a pair of detectors each detect a γ-photon within a short time frame, it is assumed that annihilation took place along the line connecting both detectors, a process referred to as coincidence. Since millions of coincidences are detected during a PET scan, this information can be used to reconstruct a 3D image of the distribution of the radiotracer within the subject/object that is positioned inside the ring of detectors (i.e., the field of view or FOV). A positron-emitting nucleus can be incorporated in naturally occurring molecules, such as H_2_O or CO_2_ (Hubeau and Steppe, 2015). According to the tracer principle, these molecules are absorbed via normal metabolism and are distributed similarly throughout the study object as non-labeled molecules (Saha, 2016). Moreover, radiotracers are administered in very small concentrations (nanomolar to picomolar range) in order not to alter or perturb the system (Turkheimer et al., 2014). The combination of these properties allow PET to study biochemical processes *in vivo*, i.e., without disturbing the object under study, which is a major asset of this technique.

### Why Should We Use PET in Plant Studies?

Positron emission tomography has become one of the most common and useful imaging modalities for detection and treatment monitoring of human diseases because of its high diagnostic efficacy and accuracy (Saha, 2016). Additionally, this imaging technique is used in preclinical studies on rodents and nonhuman primates for research on drug development linked to, e.g., cardiology or neurology (Ametamey et al., 2008). Given the non-invasive *in vivo* nature of this technique, its use has been extrapolated to plant science. Whereas preclinical studies on animals and clinical trials on human subjects are governed by ethics limiting the number of individuals to be investigated, this is not the case for studies on plants which avoids considerable administration. Although the number of studies on plants is still limited, this functional imaging technique has already shown its applicability to investigate, e.g., the transport of nutrients, phytohormones and photoassimilates (Minchin and Thorpe, 2003; Kiser et al., 2008; Jahnke et al., 2009; Hanik et al., 2010; Hubeau et al., 2018). Moreover, detection of γ-photons emitted by the radioisotopes enables tracking the transport and distribution of the radiotracers in the plant as a function of time. This is a decisive advantage to study dynamic processes like, for instance, CO_2_ transport in xylem of tree branches and leaves. Studies that investigated this process with stable ^13^C-carbon (e.g., McGuire et al., 2009; Bloemen et al., 2013a, b; Boellaard et al., 2015) or unstable ^14^C-carbon (e.g., Langenfeld-Heyser, 1989) made use of measurement techniques (i.e., isotope-ratio mass spectrometry and autoradiography) that produce discrete temporal results. Although interesting data has been obtained, the results only showed tissue enrichment in a certain treatment at a given point in time after the onset of labeling. A study that applied ^11^C-carbon in combination with PET to investigate the fate of xylem-transported CO_2_ resulted in dynamic data which allowed compartmental modeling to disentangle tracer enrichment in physiological parameters characterizing this process (i.e., CO_2_ efflux rate to the atmosphere, assimilation rate by woody tissues and internal CO_2_ transport speed) (Mincke et al., 2020). Additionally, the short half-live of the radiotracers (e.g., 2 – 109 min for the most used radioisotopes in plant science – Table 1) in combination with the non-invasive nature of PET enable the same plant to be scanned multiple times without destructive sampling. This feature allows to investigate the plant’s response to environmental changes within the same plant (Kiser et al., 2008). This methodological advantage was also used to investigate photosynthate translocation from strawberry leaves into fruits. First, non-destructive ^11^C-based imaging was applied to visualize photosynthate transport, and destructive ^13^C-labeling was applied afterwards on the same plant to quantify photosynthate content (Hidaka et al., 2019). Additionally, a study that investigated the effect of girdling on phloem transport dynamics was able to reuse the same young oak trees before and after girdling for up to five measurements in 1 week (De Schepper et al., 2013a). It was found that the position and speed of phloem transport in stems (with a diameter of 1 cm) changed after complete or partial girdling, a result that could only be obtained due to the non-invasive nature of PET. Furthermore, PET is especially suited to decipher phloem functioning. Since this tissue type is pressure-driven (De Schepper et al., 2013b), it is easily disturbed through transport or displacement, complicating its investigation (Pickard and Minchin, 1990; Turgeon and Wolf, 2009). Radiotracers enable visualization of the sugar flow without damaging or perturbing phloem transport. As such, dynamic positron-based imaging has successfully been used to investigate photosynthate translocation to storage organs like, e.g., root crops or fruits (Jahnke et al., 2009; Kawachi et al., 2011; Yamazaki et al., 2015; Hidaka et al., 2019; Kurita et al., 2020), phloem vulnerability to drought (Hubeau et al., 2018), and the effect of electric shock and cold shock on phloem transport (Pickard et al., 1993). Besides studies on phloem functioning, positron-based imaging has also been used to study the transport of jasmonate (i.e., a signal metabolite involved in plant defense) in whole plants (Ferrieri et al., 2005; Thorpe et al., 2007) as well as nitrate transport (Kiyomiya et al., 2001b; Kawachi et al., 2008). An overview on transport of plant metabolites using positron emitting isotopes is given by Kiser et al. (2008); Hubeau and Steppe (2015), Schmidt et al. (2020).

**TABLE 1:** Production information and potential tracers of positron emitting tracers used in plant science along with their half-life.

**Radio-nuclide**	**Target material**	**Nuclear reaction**	**Potential tracers for plant experiment**	**Half-life (min)**
^11^C	N_2_ + 5% H_2_ N_2_ + 0.1% O_2_	^14^N(p,α)^11^C	^11^CO_2_, ^11^C-methyl jasmonate	20.36
^18^F	H_2_^18^O	^16^O(p,n)^18^F	2-^18^F-fluoro-2-deoxy-D-glucose (^18^FDG) ^18^Fluorine (aq.)	109.74
^13^N	H_2_^16^O	^16^O(p,α)^13^N	^13^N${\text{O}}_{3}^{-}$, ^13^N_2_, ^13^NH_4_	9.96
^15^O	N_2_	^14^N(d,n)^15^O	H_2_^15^O	2.03

Nevertheless, the full potential of ^11^C-PET in plant studies remains largely unexploited. Unlike human or laboratory animal imaging, where the object size is fairly fixed, the size of plant tissues may range from several millimeters to one meter, indicating that the scanner should have a large field of view (FOV) and a high spatial resolution. However, most of the PET studies carried out on plants use either PET systems that were specifically developed for plant imaging (Kume et al., 1997; Uchida et al., 2004; Jahnke et al., 2009; Beer et al., 2010; Wu and Tai, 2011; Weisenberger et al., 2012; Wang et al., 2014) or laboratory animal PET scanners (e.g., Alexoff et al., 2011; Hubeau et al., 2018), which are both characterized by a limited FOV (axial and transverse FOV of *∼* 7 and 10 cm, respectively, for cylindrical detector configurations as depicted in Figure 1, or *∼*13 × 20 cm for planar detector configurations). Although these scanning systems benefit from a high spatial resolution (*∼*1.5 mm and sometimes submillimetre) generally only one or two plant organs (stem, leaves, fruits, or roots) can be visualized (e.g., Jahnke et al., 2009; Hubeau et al., 2018; Hidaka et al., 2019). Additionally, a more comprehensive view of whole-plant carbon allocation patterns can be gained from mature organs in large plants, where a quasi-active carbon sink for carbohydrate storage competes with different plant carbon sinks as growth or respiration (Sala et al., 2012; Hartmann and Trumbore, 2016). These difficulties may be overcome by making use of clinical PET systems, which are developed for human imaging, as these systems have two main advantages. Firstly, these imaging devices allow visualization of larger objects since they are characterized by a transverse and axial field of view (FOV – Figure 1) up to 85 and 26 cm, respectively (Vandenberghe and Marsden, 2015; Vandenberghe et al., 2016). Additionally, clinical PET scanners are equipped with a moving bed on which the plant can be placed, which enables visualization of even larger plants than the volume of the FOV, by acquiring multiple bed positions that can be stitched together into a larger volume. Another advantage of clinical PET systems is that they are nearly exclusively used in combination with structural imaging like computed tomography (CT) or magnetic resonance imaging (MRI). Consequently, the functional information provided by PET can be combined with structural data provided by CT or MRI, but only few plant studies have been reported making use of this multimodal imaging approach (e.g., Jahnke et al., 2009; Garbout et al., 2012). A drawback of clinical PET systems is the lower spatial resolution (*∼*3 – 5 mm - Vandenberghe and Marsden, 2015) compared to the laboratory animal PET scanners (España et al., 2014; Fine et al., 2014). A poor spatial resolution implies that small plant tissues cannot be distinguished from each other on the resulting PET images, e.g., phloem from xylem in small branches or different parts of a fruits’ pericarp or seeds. However, a good resolution is not always mandatory which is the case when long-distance transport of the radiotracer (in the order of 10 cm) is intended, e.g., transport of photosynthates from leaf to fruit or phytohormone transport. Additionally, the FOV of clinical PET systems have a horizontal axis while in some cases where large plants are studied, it might be appropriate to have a vertical orientation of the PET scanner. An overview of the above-mentioned specifications of laboratory animal and clinical PET imaging systems is given in Table 2 along with those for an ideal plant-PET system. Additionally, environmental parameters within the PET room that are of relevance for plant science, are listed. Note that temperature and relative humidity within a PET room are tightly controlled by air-conditioning. Lighting providing photosynthetically active radiation (PAR) is not present inside a PET room but the FOV is generally spacious enough to include LEDs beside the plant material.

**TABLE 2:** Generalized specifications of laboratory animal and clinical PET imaging systems as well as for the ideal plant-PET system. Additionally, environmental conditions within the PET room, which are of relevance in plant science, are listed.

		**Laboratory animal PET**	**Clinical PET**	**Ideal plant-PET**
**Specifications of PET imaging system**				
FOV size [cm]	Axial	7 – 12	16 – 26	Depending on the plant structure of interest
	Transverse (diameter)	10 – 12	70 – 90	
Max. object length to be scanned due to moving bed* [cm]		∼ 25	160 – 190	cm – m, depending on the plant species
Axial orientation of FOV		Horizontal	Horizontal	Switchable, depending on plant orientation
Spatial resolution [mm]		0.85 – 1.5	3 – 5	As small as possible
Sensitivity [%]		7 – 12	3 – 5	As high as possible
**Environmental conditions within PET room**				
Room temperature [°C]		∼ 18, controlled by air-conditioning	∼ 18, controlled by air-conditioning	Same as growth conditions^†^
Temperature inside FOV [°C]		Up to ∼37 by bed heating which is commonly available	Room temperature	Same as growth conditions^†^
Relative humidity [%]		∼ 40, controlled by air-conditioning	∼ 40, controlled by air-conditioning	Same as growth conditions^†^
PAR availability		Not present by default	Not present by default	In- and outside of FOV

*No dynamic tracer studies are possible when bed position changes during scanning.

*^†^Can be obtained by enclosing the plant (tissue) in a bag or plexiglass system.*

Despite the intensive occupancy of clinical PET systems, we believe that studies making use of these functional imaging devices will make an important contribution to reveal complex *in vivo* interactions in plants, like the link between xylem and phloem tissue. For example, dynamic PET imaging in combination with compartmental modeling could potentially be applied to investigate phloem vulnerability to drought by repeatedly labeling a tree that is gradually experiencing more drought stress. The same combination of dynamic PET and modeling can be employed to investigate whether xylem embolism repair relies on photosynthates that originate from phloem, storage or local production related to woody tissue photosynthesis. Furthermore, improving our understanding of the mechanisms that drive phloem transport will undoubtedly lead to new approaches for manipulating photoassimilate allocation patterns in crops and fruits.

## Experimental Design of Plant-Pet Studies

**FIGURE 2:**
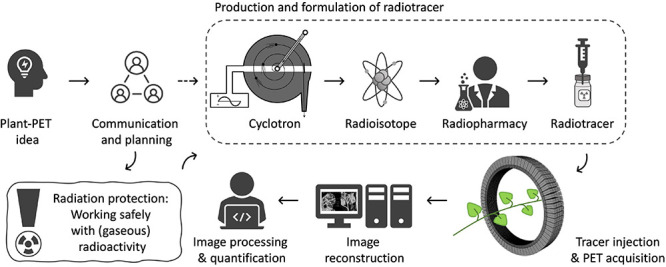
Schematic showing the multidisciplinary steps in performing PET experiments on plants.

The objective of PET imaging is to acquire (quantitative) images of the distribution of a certain radiotracer in the object under study. To obtain these images a multidisciplinary trajectory is followed within a PET center (Figure 2) of which the experimental setup is typically composed of six parts. It starts with (i) making contact with a PET center to communicate and discuss the researchers’ innovative plant-PET ideas. Prior to the execution of (test) experiments (ii) radiation protection should be discussed thoroughly to both minimize exposure to ionizing radiation and achieve conformity with the internal policy of the facility. The next step takes place in a radiochemistry lab or radiopharmacy department and involves cyclotron production of the positron emitting isotope, subsequent radio-synthesis (to obtain the desired radioactive molecule), purification and formulation. The radiotracer is then transported to the (iii) PET scanner. Due to the short half-life of PET isotopes (see Table 1), the PET scanner and cyclotron units are generally in close proximity. The PET system is typically operated by a high-level technician or researcher, while a medical physicist keeps track of the quality assurance of the PET system. Visualization of the acquired PET data is realized through (iv) mathematical reconstruction algorithms, which are generally included in PET imaging software. Once 3D images are obtained, (v) image analysis and quantification can take place.

### Communication and Planning

The basic requirement for conducting plant-PET experiments is access to both radioisotopes and a PET scanner. If not yet the case, contact should be established with key staff of a PET center, i.e., a medical imaging expert (e.g., typically the head of the preclinical imaging lab or clinical PET center) and a radiochemist, at least, if the PET center is accommodated with a cyclotron to produce the required radiotracer. PET centers are growing in large numbers worldwide and can be found in academic institutes as well as in smaller and larger hospitals. Smaller hospitals usually do not have a cyclotron, generally have a single PET scanner (typically combined with CT) and purchase their PET radiopharmaceuticals from commercial vendors that have a cyclotron facility. Larger hospitals and academic institutes have PET centers that can accommodate one or more cyclotrons, a radiochemistry laboratory and often several (multimodal) PET scanners, including laboratory animal (e.g., Alexoff et al., 2011; Hubeau et al., 2019b), clinical (e.g., Garbout et al., 2012; Karve et al., 2015), or self-designed imaging systems (e.g., Uchida et al., 2004; Jahnke et al., 2009; Weisenberger et al., 2012; Kurita et al., 2020). In these larger centers the integral multidisciplinary workflow (Figure 2) can be followed. As indicated earlier, the most frequently used PET isotopes in plant science are characterized by a short half-life (2.03, 9.96, 20.39 min for ^15^O, ^13^N and ^11^C, respectively - Table 1) making it necessary for them to be produced on site. An exception is the longer-lived ^18^F (half-life 109.74 min), which can be purchased from an isotope supplier, e.g., Curium (France & United States), NTP (South Africa), Isotope-Rosatom (Russia), and ANSTO (Australia). Purchasing radiotracers is regulated and can, for example, only be done via a hospital’s radiopharmacy. There are more than 700 cyclotrons available worldwide of which many are dedicated to the production of PET isotopes (IAEA, 2012). Most of the recent cyclotron facilities are primarily constructed for the production of ^18^F in the form of the well-defined radiotracer ^18^FDG (2-[^18^F]-fluoro-2-deoxy-*D*-glucose) for cancer detection. Additionally, a sizeable fraction of these facilities has active research programmes for the creation of other ^18^F-labeled compounds and ^11^C-labeled compounds (IAEA, 2012). Hence, there is a high probability that one of the nearest cyclotron departments is able to produce ^11^C and potentially ^11^CO_2_, subject to some changes (see “Production and Formulation of Radiotracers”). Assuming that the PET center is interested in a mutual cooperation and a cyclotron facility is able to deliver the required tracer, proof of concept experiments may be organized to investigate the feasibility of the proposed plant-PET idea.

Other important points of discussion are related to the provisioning of dedicated lighting and space. Because of the strict regulations regarding radiation exposure, PET centers are heavily shielded to minimize radiation exposure to workers (Saha, 2016). This usually implies that the rooms do not have windows and thus have a limited availability of sunlight. By consequence, it is advised to provide (timed) lighting supplying PAR to maintain regular plant functioning when performing plant-PET imaging. Additionally, depending on the size of plant species that will be investigated, it might be necessary to discuss the availability of sufficient space to (safely) store plants before and after scanning. Lastly, due to the seasonal dependence of plant material, it is advised to plan experiments well in advance (*∼*2 months, although depending on the number of scans) as these medical imaging devices are generally well occupied.

### Radiation Protection: Working Safely With (Gaseous) Radioactivity

Performing experiments with PET tracers involves exposure to ionizing radiation which could lead to harmful effects. To measure the amount of and exposure to ionizing radiation, several units are used. As indicated earlier, radioactive decay of a PET isotope occurs by the emission of a positron from its nucleus. Since this is a dynamic process, the amount of radioactivity of this type of radiotracers (as well as others used in e.g., SPECT) is quantified by the number of nuclei that decay per unit time. The standard international unit of radioactivity is Becquerel (Bq). One Bq corresponds with one disintegration per second. Curie (Ci) is the original unit of radioactivity and corresponds with the amount of radiation that is produced by one gram of radium (^226^Ra). This is an enormous unit as it equals 37 GBq compared to clinical used activities for PET imaging, which are in the range of 37 – 740 MBq (1-20 mCi). Regardless the amount of activity used, radiation exposure should be reduced or preferably avoided at all time, which forms the basis of radiation protection. This can be realized by using protective measures, personally and set-up wise.

#### Personal Radiation Safety

Concerning personal safety, it is of interest to quantify the radiation energy absorbed by biological tissues, i.e., “absorbed dose” as well as to evaluate the harmful effect of a radiation dose to an organism, i.e., “effective dose.” Both PET and computed tomography (CT) can lead to exposure to ionizing radiation. For PET imaging, annihilation generates two γ-photons, each having an energy of 511 keV. As a reference, this energy is higher than the energy of X-rays that are produced in computed tomography (CT) (20 – 130 keV) to create anatomical images or “slices” of specific areas of the body. Given the high energy γ radiation of PET tracers and the relatively small dimensions of plant tissues compared to humans, virtually all of the photons will escape plant tissues so that both absorbed and effective dose are not common for plant tissues. However, they are of importance for the researcher working with radioactivity. Working with ionizing radiation requires wearing a dosimeter badge that monitors the cumulative absorbed radiation dose. Several types of dosimeters exist, i.e., with and without live readout. A dosimeter is typically worn at chest-level on the outside of clothing and generally represent the exposure to the whole body. The absorbed dose is used to calculate the effective dose which takes into account the radiation type (α, β, or γ radiation) and the radio-sensitivity of the exposed organ (Turkheimer et al., 2014; Lakhwani et al., 2019). The limit on effective dose for occupational exposure (e.g., researchers performing PET studies) is regulated and should not exceed 20 millisievert (mSv) per year averaged over five consecutive years and of 50 mSv in any single year (IAEA, 2018). As a reference, cosmic ray exposure of a person in a jet aircraft with a flying time of 200 h in a year at an altitude of 12 km is approximately equivalent to an annual effective dose of about 1 mSv.

Furthermore, exposure to radiation should be minimized according to the triad of “Time-Distance-Shielding” (Lakhwani et al., 2019). Each factor has a different impact on the absorbed dose. Time is related to the exposure opportunities to a source of radioactive radiation as well as the time of exposure, and it is obvious that these should be reduced. Additionally, radiation exposure is inversely proportional to the square of the distance from the source. This means that doubling the distance reduces the exposure to one quarter. For example, the cumulative exposure a radiation worker receives from a 555 MBq (15 mCi) ^11^C source while standing for 2 h at a distance of 1 m instead of 2 m goes from 50.02 to 12.5 μSv (calculation see Supplementary File 1). Therefore, although strongly depending on the dose and exposure time, a general distance of at least 2 m from the source of radiation may be considered safe. Furthermore, the use of shielding is most effective to reduce radiation exposure. Appropriate stopping material for γ-photons are lead or concrete and allow reduction of exposure that is exponential to the thickness of the material (Table 3; Turkheimer et al., 2014). However, γ-photons are far more energetic than X-rays so traditional protective clothing, such as lead aprons, lead goggles or lead gloves are far less effective if not useless in a PET environment. Note that most, if not all, imaging institutions require (annual) training on safe handling of ionizing radiation and on radiation protection for people exposed to it.

**TABLE 3:** The thickness of an absorbing material required to reduce the intensity or exposure of a radiation beam (in this case 1 MeV γ rays) to one-half of the initial value when placed in the path of the beam.

**Material**	**Half-value layer [cm]**
Wood	29
Packed soil	10.1
Water	9.9
Concrete	6.5
Lead	0.9

#### Experimental Radiation Safety

**FIGURE 3:**
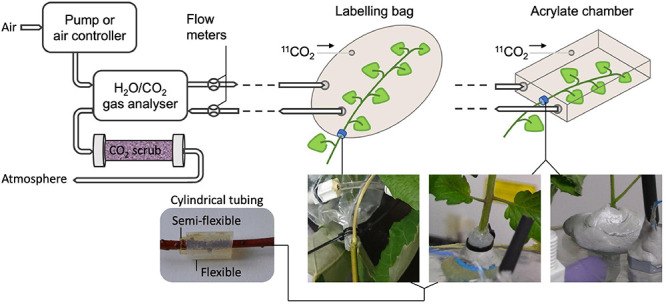
Schematic showing a potential set-up of an air circulation system of a PET experiment using gaseous ^11^CO_2_ (top) with specific examples to hermetically seal a plant part (bottom). Air flow is typically provided by a pump or another air controlling device to the plant tissue that will be labeled with ^11^CO_2_. Photosynthesis and transpiration can be obtained by a gas analyzer measuring CO_2_ and H_2_O content, respectively, of the incoming and outgoing air of the labeling system. Flow meters are used for detection of undesired leaks in the labeling system. At the end of the circulation system, the air can be scrubbed from ^11^CO_2_ before being released to the atmosphere. The bottom pictures show effective methods for enclosing part of a plant organ in a labeling bag or an acrylate feeding chamber, while hermetically sealing it from the atmosphere and other plant parts without damaging the tissue. The plant organ can for instance be enveloped by (multiple) small cylindrical flexible pieces of tubing, which are lubricated with petroleum jelly on the inside (bottom left). Straps can then be tightened upon the tubing (bottom middle) to close the labeling bag or fix the plant position in the acrylate chamber. Consecutive application of a stiff semi flexible and a soft flexible piece of tubing ensures airtightness without tissue damage when cable ties are tightened. Alternatively, malleable polysiloxan material can be used (bottom right).

Regarding the set-up for experiments with ^11^CO_2_, extra attention should be paid since this radiotracer is a gas under standard temperature and pressure. Therefore, airtightness must be achieved and maintained throughout the entire experiment to reduce the risk of radioactive gas being released into the atmosphere. However, this is challenging since plants require continuous supply of CO_2_ to maintain photosynthesis. Therefore, most systems enclose the plant, or part of it, in a labeling chamber that is connected to a gas circulation system (e.g., Kawachi et al., 2011; Dirks et al., 2012; Agtuca et al., 2014; Hubeau et al., 2018; Figure 3, upper part). A straightforward method to detect leaks in the labeling chamber is to measure the in- and outflowing air using flow meters (Hubeau et al., 2018). However, the main challenge remains to enclose the plant tissue in an airtight way. When studying or labeling a photosynthesizing organ, the labeling chamber usually has to be made out of translucent material (e.g., plexiglass or see-through plastic) to allow illumination of the plant material with PAR. Airtight constructions enclosing an entire plant can easily be made of acrylate (e.g., Karve et al., 2015), whereas enclosing a plant part (e.g., leaf or branch) can be done using both an acrylate chamber (e.g., Plexiglass, Kawachi et al., 2011), or plastic bag (e.g., Hubeau et al., 2018). When enclosing only a part of the plant, damaging the tissue should be avoided to not disturb plant functioning. An elegant way is to envelop the plant tissue with one or multiple concentric cylindrical pieces of flexible tubing (Figure 3, bottom left) which are lubricated on the inside with petroleum jelly (e.g., Vaseline^®^). Applying petroleum jelly in a syringe makes it convenient to apply it to the cylindrical tubing. The labeling chamber can then be closed by using small straps or cable ties for both a bag and an acrylic feeding cell (Figure 3, bottom middle) over the tubing without pinching off the phloem and xylem tissue to maintain regular sugar and water transport, respectively. Enclosing the plant tissue first in a somewhat stiff (i.e., semi-flexible) piece of tubing offers good protection to prevent damage when tightening the cable ties. Applying a second piece of soft tubing ensures airtightness of the system. Alternatively, malleable polysiloxan material (e.g., Terostat-IX, Henkel AG & Company, KGaA, Düsseldorf, Germany – Figure 3, bottom right) can be used to separate a plant tissue from other plant parts and the atmosphere. Zipper (storage) bags are elegant to be used as labeling bag because they come in different sizes and allow to reposition the plant tissue after enclosing the bag around the plant tissue. Modifying the shape of a labeling (zipper) bag can easily be done using a vacuum sealing device. A drawback of using small labeling chambers is the difficulty to control the microclimate, especially relative humidity tends to be higher at lower air flows (i.e., lower air renewal rate) due to transpiration of the plant tissue. To avoid ^11^CO_2_ that is not taken up by the plant to enter the atmosphere, the outflowing air system can be connected to a CO_2_ scrubbing column (Figure 3), containing soda lime pellets (Hubeau et al., 2018). In turn, the scrubbing column can be shielded with chevron lead bricks, which are commonly available in a PET center or imaging lab. Internal policy regarding radiation safety on the experimental site may require drafting a standard operating procedure (SOP) to assure safe practice and a risk analysis (RA) to indicate and assess risks of executing experiments with new tracers (e.g., ^11^CO_2_). It is hereby recommended that airtightness of the experimental set-up can be checked at any time during the experiment so that, when a leak is detected while the activity is still high, evacuation can take place.

### Production and Formulation of Radiotracers

Whereas nowadays radioactive tracers are inherently linked to clinical practice, their first application to study biological processes made use of plants and was described by de Hevesy (1923). He played a key role in the development of radiotracers, which has indirectly led to the development of nuclear medicine and PET imaging. As indicated earlier, the production of radiotracers for PET imaging starts with a cyclotron, where a charged particle (usually a hydrogen ion, e.g., H^+^) is accelerated to a high velocity to bombard a target atom, eventually creating an unstable nucleus that decays by positron emission. Depending on the target atom a certain radionuclide can be produced (Table 1). The most widely used positron-emitting nuclide in plant science is carbon-11 (^11^C, half-life of 20.39 min), which is usually administered as gaseous ^11^CO_2_ to study long-distance transport of photosynthates (Minchin and Thorpe, 2003; Karve et al., 2015; Hubeau et al., 2018) or can also be administered in an aqueous solution to study xylem-transported CO_2_ (Bloemen et al., 2015; Mincke et al., 2018, 2020; Hubeau et al., 2019b). ^11^CO_2_ is generally produced in two different ways depending on the target material, i.e., N_2_/O_2_ (Karve et al., 2015) or N_2_/H_2_ (Hubeau et al., 2018). In the former case, the nuclear reaction results immediately in the formation of ^11^CO_2_, however, with the undesired by-product ^11^CO. Yet, CO can be oxidized to CO_2_ by passing the target gas over hot copper oxide (Ferrieri and Wolf, 1983; Saha, 2016). Application of N_2_/H_2_ results in the formation of ^11^CH_4_ which subsequently needs to be oxidized via a cobalt oxide column to yield ^11^CO_2_ (Landais and Finn, 1989). This last step involves heating to 500 °C, requiring the use of a tube furnace that might not be a part of the standard equipment in a cyclotron unit. Other, albeit less frequently used, methods to produce ^11^CO_2_ are described by Ferrieri and Wolf (1983). Guidance information on operation and maintenance together with methodologies and relevant analyses regarding cyclotron production of the radionuclides listed in Table 1 is presented by IAEA (2012), which can be downloaded for free from the IAEA website along other complementary information regarding the development and production of radioisotopes and generators.

After its production, ^11^CO_2_ can either be channeled immediately to the plant labeling chamber when a direct connection is made from the cyclotron target or be trapped in a portable medium to be transported to the PET scanner. In the former case, ^11^CO_2_ can be concentrated through selective adsorption onto a molecular sieve (Ferrieri et al., 2005; Babst et al., 2013). Once the tracer is concentrated, it can be desorbed from the module to be directed to the experimental labeling chamber using a controlled air flow (Ferrieri et al., 2005). When ^11^CO_2_ requires transport to the nearby PET facility it can, depending on the research objective, either be trapped in a NaOH solution to be applied as a gas (e.g., Hubeau et al., 2018), or bubbled through a slightly acidic buffer (e.g., Tris, phosphate or citric acid) to obtain an aqueous ^11^CO_2_ solution (e.g., Mincke et al., 2018). In both cases, the liquid tracer solution can be transported in a shielded syringe carrier. The dissolved ^11^CO_2_ can be released from the NaOH solution by injection into an excess acidic solution (e.g., H_2_SO_4_), which can subsequently be directed towards the plant tissue by bubbling air into the solution. Safe transport of gaseous ^11^CO_2_ trapped in a miniature molecular sieve of a portable handheld delivery system (Kim et al., 2014) or a stainless steel trap immersed in liquid nitrogen or liquid argon (Ishioka et al., 1999; Hidaka et al., 2019) are described. With regard to the formulation of a ^11^CO_2_-enriched buffered solution that has to be exposed to the xylem (regardless the radioisotope), the buffer’s pH is allowed to deviate slightly from the pH of xylem sap of the species under study. Specifically, once the tracer is taken up, equilibrium reactions will occur, creating the right pH inside the tissue (Butler, 1991). Hence, the pH of an ^11^CO_2_-enriched aqueous solution can be slightly more acidic than the xylem sap to favor the ^11^C-label being dissolved as CO_2_ (aq.) over bicarbonate.

The use of ^11^C is not limited to CO_2_ as it can also be built into other traces like methyl jasmonate, auxin or salicylic acid (Thorpe et al., 2007; Agtuca et al., 2014). Other positron-emitting isotopes applied in plant studies are fluorine-18 (^18^F), nitrogen-13 (^13^N) and oxygen-15 (^15^O), which can be incorporated into biologically active molecules like ^18^FDG, ^13^N${\text{O}}_{3}^{-}$ and H_2_^15^O, respectively. Therefore, the use of these radiolabelled molecules includes, but is not limited to, investigating sugar transport (e.g., Fatangare and Svatoš, 2016), nitrate uptake via roots (e.g., Siddiqi et al., 1989; Liang et al., 2011), and water transport (e.g., Mori et al., 2000; Kiyomiya et al., 2001a), respectively. Application of ^18^F-fluorine is described as a proxy for tracing water transport (e.g., Ishioka et al., 1999). ^13^N has also been applied as gaseous ^13^N_2_ to study nitrogen fixation of rhizobium root nodules (Ishii et al., 2009; Kasel et al., 2010; Yin et al., 2019) and as ammonium (^13^NH_4_) to study the effect of nitrogen deficiency, phytohormones and lighting treatments on its uptake and translocation in rice plants (Kiyomiya et al., 2001b). A nice tabular overview of positron-based plant experiments carried out to date, including the topics listed above as well as uptake and translocation of heavy metals, is provided by Schmidt et al. (2020). The above-mentioned radiotracers, together with their involved pathways, form only a fraction of the potential molecules that can be studied in plants using PET imaging. By making use of organic (radio)chemistry, radionuclides can be incorporated in many other dedicated molecules. However, due to the short half-life, the isotope needs to be labeled to the required molecule by a radiochemist in a short time frame requiring simple and efficient chemical conversion and purification methods (Figure 2). After labeling, the radiotracer is ready to be exposed to the plant material and PET imaging can start.

### Pet Data Acquisition

#### Scan Time

An important issue with PET imaging is the restriction on the experiment’s acquisition or scan time. The radionuclide’s half-life is hereby one of the main determining factors and should be considered together with the final radioactivity of the formulated tracer that is ready to be exposed to the plant tissue. Higher activities enable possibilities to longer scan times, although it should be noted that the allowed radioactivity that can be brought into an imaging facility is likely to be regulated and limited. For studies using ^11^C (half-life 20.39 min), typically 111 – 740 MBq (3 – 20 mCi) is used in which the plant tissues can be scanned for ∼ 2 – 3 h (note that the activity needs to be doubled to scan another half-life longer). Scan times for studies using ^15^O and ^13^N (both having a shorter half-life, 2.03 and 9.96 min, respectively) will be shorter whereas studies with ^18^F (half-life 109.74 min) can take longer. Other factors affecting the scan time are the time for uptake and plant metabolism as well as the dynamic range of the PET scanner. Generally, if dynamic imaging is intended, it is appropriate to use PET isotopes if the metabolic process of interest alters the tracer distribution within ten half-lives of time (Schmidt et al., 2020). For example, application of ^15^O is useful for studying fast-metabolizing kinetics such as water transport, but it is very difficult to use this isotope for studying slow-metabolizing pathways involving for instance passive processes. Additionally, the researcher should take into account that the amount of tracer supplied to the plant is generally not entirely taken up. With regard to studies using gaseous ^11^CO_2_ an uptake ratio of 80 – 90 % can be achieved by pulse labeling leaf tissues and subsequently stopping gas circulation to the labeling chamber for several minutes (e.g., 5-7 min; personal experience). The last main determinant of the scan time is the dynamic range of the positron-based imaging device. This is especially important for application of ^13^N and ^15^O. These radionuclides are characterized by a short half-life and in order to scan as long as possible, a PET scanner is required that is able to handle both high (i.e., high count-rate accuracy) and low (i.e., high sensitivity) activities.

#### Image Degrading Effects

**FIGURE 4:**
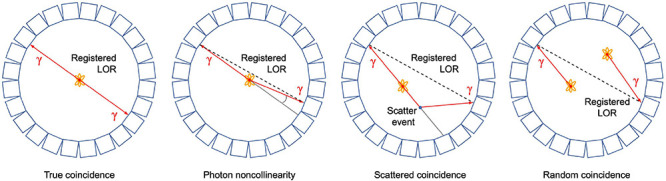
Schematic representation of a true coincidence event and several image degrading effects in positron emission tomography, i.e., photon noncollinearity, scattered and random coincidence. In each case, the resulting line of response (LOR) that is registered by the detectors is shown.

As mentioned above, PET is based on the detection of two photons (511 keV each) that originate from β^+^ emitting radiotracers (e.g., ^11^CO_2_). The two photons are detected electronically, i.e., coincidence events, using a ring of detectors (Figure 1). When two photons are detected by two different detectors from the detector ring, it is assumed that the annihilation occurred along the straight line connecting the centers of both detectors, called the line of response (LOR). During a PET scan millions of LORs are detected that are used to reconstruct an image of the *in vivo* distribution of a radiotracer. Despite this simple concept of positron imaging, different factors can degrade the image obtained by a PET scanner due to physics or system performance. These effects include photon noncollinearity, scattered coincidence and random coincidence (Saha, 2016) and are shown schematically in Figure 4. Whereas photon noncollinearity implies a small error compared to both random and scatter coincidence events (especially in preclinical scanners with a small diameter), both the latter result in the formation of a LOR that does not reflect the true location of annihilation and thus degrades image quality. A higher ratio of the true-to-scatter/random coincidence events may improve PET system performance. Such considerations led to the development of the noise equivalent count rate (NECR) as a metric of PET system performance (Strother et al., 1990; Yang and Peng, 2015). Conventionally, NECR is measured for clinical PET systems by scanning the cylindrical NEMA phantom (20 cm diameter × 70 cm long) but it can be measured for laboratory animal PET systems as well by making use of a cone-shaped phantom (NEMA, 2012; Prasad and Zaidi, 2012). However, the most challenging image degrading factor with regard to imaging plants is related to the positron range. This is the distance that the positron travels through the object to lose enough kinetic energy before annihilation takes place (black zigzag pattern in Figure 1). The mean distance between decaying nucleus and the site of annihilation (i.e., mean positron range - *R*_*mean*_) for common types of radionuclides used in plant science is generally larger than 1 mm and can amount to maximum (*R*_*max*_) 4.2 mm for ^11^C (Table 4 – Conti and Eriksson, 2016), which poses challenges upon imaging leaves whose thickness is in the range of tens of μm for mesophyll and hundreds of μm up to some mm for veins (Witkowski and Lamont, 1991). Particularly, leaves of most plants are so thin that a large fraction of positrons emitted from PET isotopes escape the tissue before annihilation. Alexoff et al. (2011) found that the fractions of positrons that escaped the leaf parenchyma of tobacco plants (200 – 250 μm) were 64 ± 4%, 59 ± 1% and 67 ± 2% for ^11^C, ^18^F and ^13^N, respectively. Because the probability of annihilation increases with thickness, escape fractions were lower in thicker leaf areas like the midrib (1 – 2 mm) (Alexoff et al., 2011). When studying single leaves, an approach to increase the detection of positrons actually annihilating inside the plant material includes the use of thin acrylate plates that can be positioned parallel with the leaf blade, while ensuring not to limit air contact with the leaf (Alexoff et al., 2011; Hubeau et al., 2019b). An alternative is calculating the annihilation probability of positrons according to the thickness of the tissue in which the positrons were detected. Specifically, Jodal et al. (2012) fitted an empirical equation to the cumulative annihilation probability distributions of several positron-emitting isotopes in water. Since plants mainly consist of water, the empirical equation can be used for plant tissues. Additionally, it was indicated that the annihilation probability distributions in other media were very similar to water (Jodal et al., 2012). The empirical equation is in function of the distance from the point of positron emission, and can be used to calculate the probability of positron annihilation for certain tissue thicknesses. In this way activities in plant tissues of different thickness can be normalized and compared. As a reference, the empirically calculated annihilation probability of positron-emitting isotopes commonly used in plant studies within 1 mm of water is given in Table 4.

**TABLE 4:** Mean and maximum positron range (*R*_*mean*_ and *R*_*max*_, respectively) of radionuclides commonly used in plant science along with the probability of annihilation (*P*_*annihilation*_) within 1 mm of water.

**Radionuclide**	***R*_*mean*_ (mm)**	***R*_*max*_ (mm)**	*P*_*annihilation*_ within 1 mm of water [%]*
^11^C	1.2	4.2	56.3
^18^F	0.6	2.4	83.5
^13^N	1.8	5.5	42.7
^15^O	3.0	8.4	26.7

*P_*annihilation*_ is empirically calculated according to Jodal et al. (2012).

#### Detector Configuration

Whereas clinical and preclinical PET scanners typically have a ring of detectors (Figure 1) also planar arrangements of PET detector modules are possible. A planar position of detectors results in 2D images compared to 3D images obtained by circular PET modules or several pairs of planar PET modules. Both planar (2D) and 3D PET setups have their (dis)advantages. Generally, 3D PET is the method of choice in studies where high sensitivity is required, especially when distinction between different tissues in small species is necessary, and where a lot of counts are lost because of attenuation, e.g., in thicker tissues. Additionally, 3D PET can be performed with a lower injected dose, or a reduced scan duration to a comparable planar 2D study (Dhawan et al., 1997; Saha, 2016). When anticipating dynamic PET imaging, which requires time frames of several seconds or minutes, 3D PET is put forward because of the increased sensitivity. However, 3D PET leads to an increased extent of random and scatter incidents compared to planar 2D PET (Gundlich et al., 2006; Saha, 2016). A benefit of planar PET modules is that they allow to position plant tissues freely between detectors which can, additionally, be scanned in a vertical position (Kawachi et al., 2006; De Schepper et al., 2013a). Contrarily, the circular PET modules of laboratory animal and clinical PET scanners are generally horizontally oriented, and it should be taken into account that distally located plant tissues (e.g., ramifications, roots) from the plant tissue that will be scanned (e.g., main branch, stem) need to fit through the detector ring as well. Horizontal orientation of the detector ring might require laying a plant horizontally. Whereas this may affect plant function over the long-term, an ^11^CO_2_-based study on maize observed little or no effect of horizontal positioning in terms of photoassimilate transport speeds, ^11^C fixation, or photosynthetic CO_2_ exchange rates (measured with an IRGA) compared to vertical plants within a 3 h time frame (Karve et al., 2015). It is therefore safe to assume normal plant functioning when adopting a horizontal plant position for a limited (scan) time. Additionally, most, if not all, 3D PET systems (both clinical and preclinical) are equipped with a bed that can move into the FOV as desired. This makes it possible to scan tissues larger than the axial FOV size (i.e., up to ∼25 cm and ∼190 cm for preclinical and clinical scanners – Table 2). Moving the bed during the acquisition may change the spatial configuration of leaves or other plant tissues which could create movement artifacts. Additionally, no dynamic tracer studies are possible when the bed position changes during scanning. In most preclinical scanners, the bed can be removed to gain extra space inside the FOV.

#### Complementary Measurements and Other Considerations

Relative comparison or quantification of different measurements often requires normalization based on tracer uptake by the plant tissue. If one plant tissue is to be labeled with, e.g., ^11^CO_2_, a PIN diode gamma radiation detector (e.g., Bioscan, Inc., Washington, DC, United States) can be fitted into the labeling chamber to additionally measure the amount of ^11^C-radioactivity in the labeled plant tissue (Ferrieri et al., 2005; Hanik et al., 2010; Babst et al., 2013). The data of this detector shows the detected activity over time which contains information about the amount of radioactivity administered to the plant tissue, the amount fixed by photosynthesis and the rate of export of radioactivity away from the administration zone. When gaseous ^11^CO_2_ is delivered to the labeling chamber by bubbling an ^11^C-enriched NaOH solution through an acidic solution, there is an alternative way to obtain the amount of radioactivity delivered and fixed by the plant tissue. This requires measurement of (i) the radioactivity of the ^11^C-enriched NaOH solution before injection in acid from which both, (ii) the remaining activity in the neutralized solution of NaOH and acid that was not injected in the labeling chamber, and (iii) the radioactivity that was collected by the ^11^CO_2_ trap after the experiment (Figure 3) should be subtracted. These measurements can be performed by a dose calibrator or Geiger counter which are by default available in a PET center and needs to be recalculated to one point in time before subtraction. Eventually, the amount of carbon fixation can be used to normalize and thus compare different measurements.

Another possible advantage of PET is the complementarity with positron autoradiography. After the PET experiment the plant tissue is hereby exposed to an imaging phosphor plate for typically 10-15 min, depending on the remaining radioactivity in the plant tissue. Since autoradiography requires close contact between the plant tissue and the imaging plate, these should be pushed close together. Therefore, this method is generally considered as destructive. However, the resulting 2D image gives a high-resolution snapshot in time showing the integrated tracer activity detected during the exposure time in the plant tissue. Positron autoradiography has a much higher spatial resolution (24 pixels mm^–1^) compared to PET (*∼* 0.3 – 1 pixel mm^–1^ for PET). In this way, this technique has been used to acquire detailed tracer distribution in leaves to characterize phloem loading strategies in different plant species (Hubeau et al., 2019a). Because of its high spatial resolution, autoradiography was also used to assist 2D positron-based imaging in the visualization of ^13^N-translocation in rice (Kiyomiya et al., 2001b), [^11^C]methionine (Nakanishi et al., 1999), ^52^Mn (Tsukamoto et al., 2006), and ^52^F (Tsukamoto et al., 2009) translocation in barley. Additionally, autoradiography was used in a creative way to trace carbon partitioning to the major non-structural carbohydrates (NSC) in sorghum leaves (Babst et al., 2013). Specifically, after ^11^CO_2_ labeling leaves were extracted, and the supernatant was separated in NSC using thin layer chromatography (TLC). The TLC-plates were subsequently exposed to autoradiographic phosphor plates to determine the amount of [^11^C]-labeled sucrose, glucose and fructose.

After the PET acquisition and/or positron autoradiography, there will be some radioactivity remaining inside the plant tissue and/or labeling medium. Therefore, these are regarded as radioactive waste and can be disposed of by decay in safe storage (i.e., lead castle). Because of the short half-lives, waste from ^11^C, ^13^N, and ^15^O does not need to be stored for long-term decay in storage and can be discarded or kept for further processing at the beginning of the next day at the latest. Waste of ^18^F-labeling experiments may need to be stored for decay depending on the level of activity and the time of the day when it is stored.

### Image Reconstruction

**FIGURE 5:**
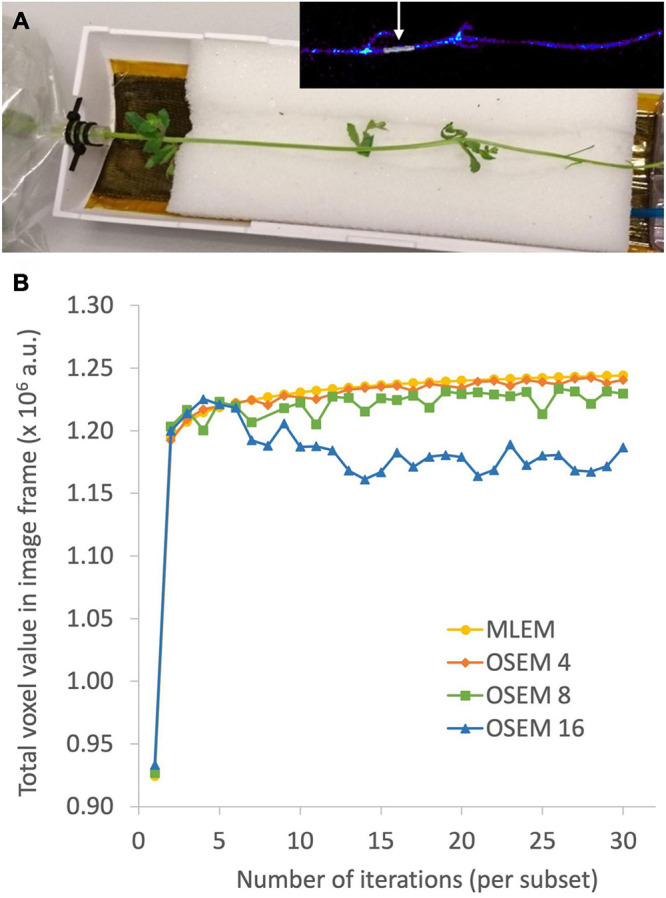
Photograph of a plant-PET setup for a study on visualizing phloem transport in Arabidopsis **(A)**. The rosette of the plant was hereby labeled with one pulse of ^11^CO_2_, while the inflorescence was positioned inside the field of view. The OSEM reconstructed time frame using 4 subsets and 30 iterations per subset is inserted in the right top corner. The region of interest, indicated by the arrow, was drawn on the reconstructed time frame and used to calculate the signal-to-noise ratio (SNR - Table 5). The effect of reconstruction algorithms MLEM and OSEM as well as the effect of a varying number of OSEM subsets X (indicated by OSEM X) on plant-PET data was investigated based on the convergence of the sum of all voxel values in the reconstructed time frame as a function of the number of iterations per subset **(B)**. Stable convergence of the total voxel value implies that more iterations will not result in a qualitatively better image, on the contrary, the noise present can be amplified with further iterations.

The aim of plant-PET is to quantitatively determine the dynamic flow of a radioactively labeled compound inside the plant. The measured data after PET scanning represent the total activity along lines of known location, i.e., LORs (Figure 4). To obtain a final image, the mathematical problem consists of reconstructing the spatial distribution of radioactivity in the plant, at specific time points from these LORs. These measurements are generally noisy because limited amounts of radioactivity will be used in practice and constraints on acquisition time due to the isotope’s half-life. For emission tomography, there are two categories of reconstruction algorithms, namely, analytical and iterative methods. The reconstruction algorithm that is used will have important effects on the noise properties of the final image. Note that regardless of the reconstruction algorithm, exponential decay of the radiotracer is corrected. However, due to the noisy nature of the acquired emission data, it is desirable to use a reconstruction approach that takes into account the statistical nature of the noise. Since the emission and detection of photons are Poisson processes, iterative methods that model Poisson statistics have become the standard for PET reconstructions (Vandenberghe et al., 2016). Analytical methods, such as the filtered back projection (FBP) algorithm, are computationally very efficient. However, they do not take into account counting statistics, and consequently the use of these analytical methods has been completely replaced by iterative reconstruction methods. Here, the maximum-likelihood expectation maximization (MLEM) is the foundational algorithm (Lange and Carson, 1984) and it has been shown that it provides images with better noise properties compared to analytical methods (Shepp et al., 1984). However, MLEM is computationally expensive and requires many iterations to reach a suitable image. To reduce computational cost, block-iterative algorithms such as the ordered-subsets expectation maximization (OSEM) algorithm (Hudson and Larkin, 1994; Hutton et al., 1997) and the Row-Action Maximum Likelihood Algorithm (RAMLA) (Browne and De Pierru, 1996; Teymurazyan et al., 2013) have been introduced and can be regarded as modified versions of MLEM and OSEM, respectively (Tarantola et al., 2003). Still, in each of these reconstruction methods the target remains maximization of a likelihood function. In both OSEM and RAMLA, the measured data is divided into subsets that are sequentially used to accelerate the reconstruction process compared to MLEM. The number of subsets provides a good estimate of the acceleration factor that can be obtained (Hudson and Larkin, 1994; Hutton et al., 1997). The effect of reconstruction algorithms MLEM and OSEM as well as the effect of a varying number of OSEM subsets on plant-PET data was tested for a study visualizing phloem transport in Arabidopsis (Figure 5A). In this study the rosette of an Arabidopsis plant was labeled with one pulse of ^11^CO_2_ while the inflorescence was positioned in the FOV. Data was acquired for 120 min but to reduce the computational cost of the reconstruction only one time frame of 5 min was selected towards the end of the scanning period, i.e., when the activity inside the FOV was maximal (inserted PET image of Figure 5A). Comparison of the different reconstruction algorithms was based on the convergence of the sum of all voxel values of the reconstructed time frame in function of the number of iterations per subset (Figure 5B). It can be assumed that with convergence of the total voxel value, more iterations will not lead to a better-quality image; on the contrary, the noise present can be amplified with further iterations. Application of MLEM and OSEM using 4 subsets (i.e., OSEM 4) led to a similar convergence of the total voxel value. The total voxel value was slightly lower when OSEM 4 was applied instead of MLEM, because each subset contained only one fourth of the acquired data. The main difference between both algorithms is the reconstruction time needed per iteration as OSEM 4 was roughly four times faster compared to MLEM (0.145 vs. 0.5 s per iteration – Table 5). Further increase of the number of subsets is accompanied with faster reconstruction speeds (Table 5) but at a cost of image quality (Figure 5B) since less data is used in each subset. An indication of image quality can be provided by calculating the signal-to-noise ratio (SNR), which was determined by dividing the average voxel value by the standard deviation in an ROI that closely fits part of the plant tissue (indicated by arrow on insert Figure 5A). As can be observed in Table 5, the SNR decreases with a higher number of subsets and one should be careful not to select too many subsets, because then each individual subset contains less tomographic and statistical information, potentially resulting in a loss of image quality.

**TABLE 5:** Comparison of reconstruction algorithms MLEM and OSEM, using different number of subsets X (indicated by OSEM X), in terms of iteration speed and signal-to-noise ratio (SNR).

**Reconstruction algorithm**	**Time per iteration [s]**	SNR*
MLEM	0.501	1.108
OSEM 4	0.145	1.113
OSEM 8	0.070	1.136
OSEM 16	0.038	1.015

*SNR was determined by dividing the mean voxel value by the standard deviation in an ROI that closely fits part of the plant tissue (indicated by arrow on insert Figure 5A). The data used for image reconstruction was one time frame of 5 min extracted from a 120-min PET scan visualizing ^11^C-phloem transport in an inflorescence of Arabidopsis (Figure 5A).

Practically, image reconstruction methods are generally included in the software that comes with the PET system. Hereby, MLEM and OSEM are currently incorporated in many PET systems and it is advised to use these reconstruction algorithms over FBP to obtain high-quality (dynamic) images. RAMLA is implemented on some commercial PET systems and, when available, could lead to faster convergence than OSEM (Saha, 2016).

Iterative methods have the theoretical potential to produce unbiased estimates of the tracer distribution within an object and thus to provide absolute quantification. Two criteria characterize the reliability of absolute quantification: accuracy and precision (Frey et al., 2012; Vanhove et al., 2015). Iterative methods can substantially improve both criteria because they include an appropriate statistical model to describe the measured data, resulting in better noise properties and thus improved precision, and they allow to accurately model image degrading effects such as photon attenuation, scattered and random coincidences (Figure 4), resulting in a more accurate representation of the tracer distribution when a sufficient number of iterations are used (Vandeghinste et al., 2014). When absolute quantification is required, it is important to perform a cross-calibration between the PET camera and the dose calibrator required to measure the amount of radioactivity used during the plant-PET experiments. Cross-calibration is a direct, relative calibration between the institution’s own dose calibrator and PET camera. In short, the procedure is as follows: a syringe has to be filled with a radioactive solution with an activity (in Bq) that is close to the injected activity applied during the plant-PET experiments. This syringe should be measured in the institution’s dose calibrator. The solution should then be introduced into a calibration phantom (mostly a cylindrical phantom) with an exact known volume (in mL) filled with water, resulting in a solution with known activity concentration in Bq/mL. After acquiring a PET scan of the calibration phantom, the acquired data have to be reconstructed using the same reconstruction parameters that will be used during the plant experiment. A region-of-interest has to be drawn on the reconstructed images of the calibration phantom in order to determine the average volumetric concentration of activity within the phantom as measured by the PET scanner. Conversion factors can then be directly derived so that the measurements from dose calibrator and PET camera can be synchronized (Boellaard et al., 2010, 2015). Karve et al. (2015) described the impact of some image degrading effects using a phantom when imaging sorghum and found that scatter correction had little effect (<1%) on the stem and shoot, whereas attenuation of the γ-photons (due to energy loss to the irradiated tissue) led to an error of 30% in the stem and 55% in the root. It is thus especially important to investigate the impact of these effects when comparing plant tissues of different sizes as well as larger tissues (e.g., stems) given the half-value layer of 29 cm for wood (Table 3). When CT data is acquired in addition to PET images, it is generally used to correct the latter for photon attenuation. CT data can additionally be used to facilitate image analysis (see “Image Processing and Quantification”).

**FIGURE 6:**
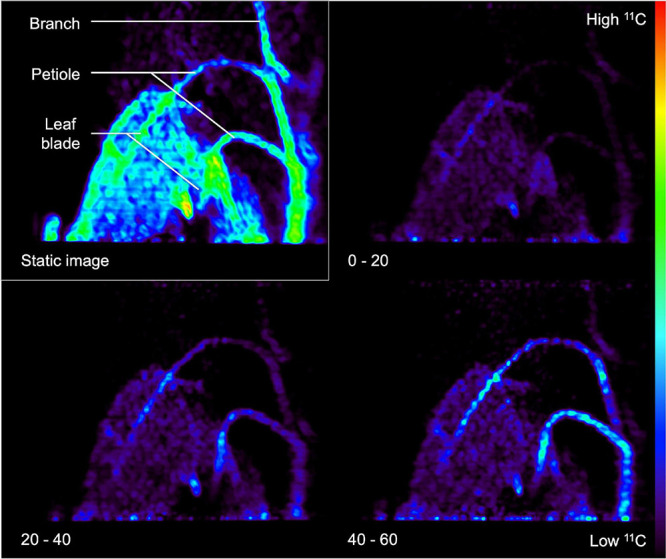
Example of a static (upper left rectangle) and three dynamic PET images (timestamp in minutes shown in the lower left corner) of a *Populus tremula* branch that was exposed to gaseous ^11^CO_2_ during a 60-min PET acquisition. Transport of the label via the petioles to the branch is visualized by dynamic PET images. The static PET image (i.e., sum of dynamic images) has a better signal-to-noise ratio and can be used for drawing regions of interest (ROIs) around the branch or petiole. These ROIs can then be copied on the dynamic PET images to obtain tracer concentrations per ROI over time, i.e., time-activity curves (TACs).

Image reconstruction is demanding in terms of computational power and time, especially when the stored LORs have to be reconstructed into different time frames to monitor a dynamic process, which is called dynamic or 4D PET. Here, series of PET images are obtained per e.g., 2–10 min of the acquisition time, depending on the sensitivity of the PET scanner and the amount of radioactivity added. However, it is also advised to reconstruct a static image that is the mean/sum of the all the individual time frames. This static 3D image has a higher SNR than the individual time frames (Turkheimer et al., 2014) and it is particularly useful for visual assessment of the entire dynamic process in one 3D image. This is demonstrated in Figure 6, where the static image is shown in the upper left corner along with some dynamic time frames of 20 min. To this end, a *Populus tremula* L. branch was exposed to gaseous ^11^CO_2_. The static image shows ^11^C-tracer accumulation in the complete branch segment inside the FOV in contrast to the dynamic images, where only part of the branch segment is visible due to dynamic nature of the process. Aside from the higher SNR, the reconstruction time of such a static image is generally much shorter than the dynamic reconstruction time.

### Image Processing and Quantification

**FIGURE 7:**
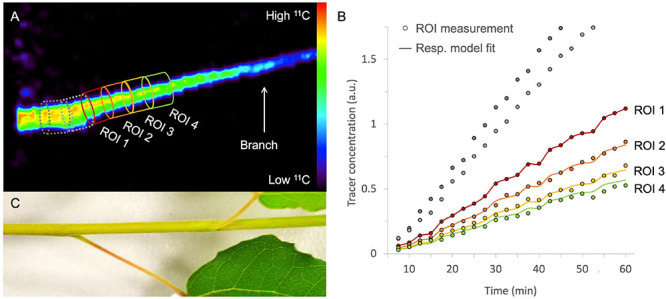
Example of a static volume rendered PET image showing xylem-transported ^11^CO_2_ in a branch segment of *Populus tremula* **(A)**. By extracting the tracer concentrations within, e.g., four consecutive ROIs of the corresponding dynamic PET images (e.g., 2.5 min time frames) time-activity curves (TACs) are obtained [circles in panel **(B)**]. Time is expressed in minutes after pulse-labeling aqueous ^11^CO_2_ to the cut end of the branch. By means of mathematical frameworks a model, representing the molecular system under study, can be fitted continuous lines) to the measured TACs. The importance of pursuing good region of interest (ROI) drawing practices is demonstrated by knowing which corresponding plant part is inside the field of view **(C)**. It would be straightforward to draw ROI 1 on the branch segment having the highest tracer concentration [dotted ROIs in panel **(A)**]. On the branch segment enclosed in these ROIs however, a petiole originates, which cannot be resolved from the branch itself due to the limited spatial resolution of the PET system. Therefore, the ^11^C-tracer detected in the branch and the petiole is added in these ROIs, resulting in an incorrect higher signal and eventually incorrect TACs [gray ROI measurements in panel **(B)**]. These ROI data sets were therefore excluded from parameter calibration and thus do not have a continuous model fit. Note that PET image **(A)** shows the side view whereas the branch **(C)** is shown from above.

After image reconstruction, while applying the necessary corrections (if needed), 3D or 4D images are obtained, which can be analyzed using image analysis software. Commonly used software includes OsiriX (Rosset et al., 2004 – commercial), Horos [^1^ GNU Lesser General Public License, Version 3.0 (LGPL 3.0) – open-source] and AMIDE (Loening and Gambhir, 2003 – open-source). These software packages allow to reduce noise by smoothing or blurring the images, which can be executed on both static and dynamic reconstructed images. A common approach is the application of a gaussian filter, whereby a gaussian curve is applied to calculate the intensity of each voxel by using a fixed number of voxels around it. However, reducing noise will also result in poorer spatial resolution. Finding the ideal trade-off between noise and spatial resolution is usually performed on the static image when a dynamic process needs to be quantified. Subsequently, the static 3D image can be used to draw regions of interest (ROIs) onto the plant tissue under study (Figure 7A). In Figure 7A, xylem-transported ^11^CO_2_ in a young branch segment of *Populus tremula* is imaged and the goal was to visualize and quantify its dynamic transport. In this example, four consecutive ROIs are drawn (colored ROIs 1 – 4) on the static 3D image because it depicts the branch more clearly than each separate image that makes up dynamic 4D image (see “Image Reconstruction”). Image analysis software allows to upload multiple datasets in one study so that the dynamically reconstructed 4D PET data can be uploaded as well. All image analysis software includes the possibility to calculate the measured activity in each ROI for any of the 4D PET images over time. This data can be plotted directly as time-activity curves (TACs – one for each ROI) which can be used for further quantification. An example of measured TACs (circles) for each of the four colored ROIs (Figure 7A) is shown in Figure 7B. TACs can for example be used to retrieve physiological properties of the plant like phloem transport speed (based on the time of first tracer arrival, e.g., Karve et al., 2015), uptake and distribution of plant nutrients like NO_3_ (e.g., Kawachi et al., 2008; Liang et al., 2011), NH_4_ (e.g., Kiyomiya et al., 2001b), or Fe (e.g., Tsukamoto et al., 2009), photoassimilate translocation to storage organs (e.g., Kikuchi et al., 2008; Hidaka et al., 2019), xylem-transported CO_2_ (Hubeau et al., 2019b), as well as changes in whole-plant carbon allocation (e.g., Karve et al., 2015).

**FIGURE 8:**
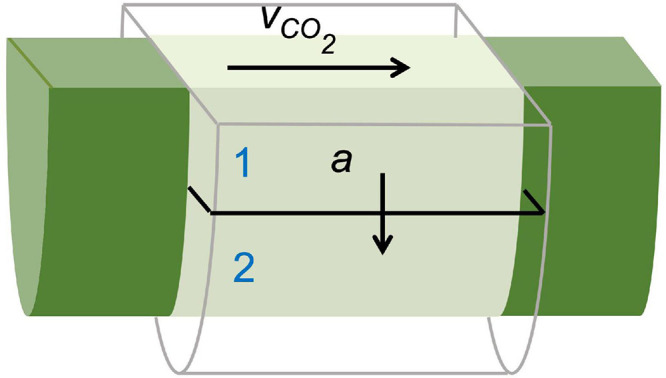
Schematic of a simplified compartmental model used to describe xylem-dissolved ^11^CO_2_-tracer movement in a cylindrical region of interest (ROI) within a branch segment shown in Figure 7A. The model is described by two parameters, i.e., xylem CO_2_ transport speed *v*_*CO*2_ (mm min^–1^) and exchange fraction *a* (min^–1^) as defined by Eqs. (S3–4) (Supplementary file 2). Through sap flow, ^11^CO_2_ enters and moves through the xylem conduits (i.e., compartment 1) of each ROI with transport speed *v*_*CO*2_. Within each ROI, ^11^CO_2_ can move from the xylem to surrounding chloroplast containing cells (i.e., compartment 2) through *a*, where it is assimilated by woody tissue photosynthesis and stored.

Additionally, dynamic PET measurements can be used as input for mathematical frameworks to retrieve physiological plant parameters that are difficult to measure with other techniques. This can be achieved by means of an input-output framework, as developed by Minchin and co-workers (Minchin and Thorpe, 2003; Minchin, 2007, 2012; Kiser et al., 2008), or by mechanistic compartmental modeling (Bühler et al., 2011, 2014, 2018; Hubeau et al., 2018). Compartmental models have an advantage over input-output models because they restrict model outcomes with physical boundaries, allowing to pose realistic ranges for solute transport characteristics (Bühler et al., 2011; Hubeau and Steppe, 2015). Therefore, compartmental models are of high interest to study long-distance transport in plants for the investigation of functional traits, especially under diverse environmental conditions (Jahnke et al., 2009). This boils down to translating the tracer dynamics (i.e., TACs) by a model that represents the system under study. The model is composed of mass balances (i.e., differential equations) defined by tracer concentrations and kinetic rate constants to describe the exchange between compartments. This method has usually been implemented with the assumption that the system under study does not change during the experiment (Minchin and Thorpe, 2003). The example of xylem-transported ^11^CO_2_ (Figure 7) is described by Mincke et al. (2020) using three compartments which will be simplified in this manuscript to a two-compartment model, purely for demonstration purposes. Each of the ROIs can be regarded as a small branch segment that is divided in two compartments, which are described by two parameters, i.e., xylem CO_2_ transport speed *v*_*CO*2_ (mm min^–1^) and exchange fraction *a* (min^–1^) (Figure 8). Sap-dissolved ^11^CO_2_ can move within xylem conduits of each ROI (compartment 1) with transport speed *v*_*CO*2_ and can move to surrounding chloroplast containing cells (compartment 2) through *a* to be assimilated and immobilized by woody tissue photosynthesis. The equations describing this model along with extra considerations on the model can be found in Supplementary file 2. This model could equally be applied to study phloem transport within a petiole or a branch after gaseous ^11^CO_2_ exposure (Figure 6) with the two parameters then being phloem transport speed and the unloading fraction.

The goal of fitting a model to dynamic tracer data (i.e., model calibration) is to derive specific parameters that have a physiological meaning, which are difficult to obtain by direct measurement. Specifically, due to the limited spatial resolution of PET (*∼* 1 - 3 mm), physiological processes in several tissues are integrated into the measured TACs. In the example of xylem-transported CO_2_, these parameters are the xylem CO_2_ transport speed *v*_*CO*2_ and the exchange fraction *a* that gets photosynthetically incorporated into the tissue. Practically, such physiological parameters can be retrieved by implementation and calibration of plant models using software packages, which include MATLAB (MathWorks, Inc, Natick, MA, United States - commercial), (R Core Team, 2020 - open source) and dedicated plant modeling software PhytoSim (Phyto-IT, Gent, Belgium - commercial). When the proposed model properly describes the system under study (i.e., TACs), model calibration should converge, resulting in the optimal model parameters. These parameters can then be used to simulate the solved differential equations which should fit the measured TACs (continuous lines in Figure 7B). When plant modeling is intended, it is advised to have a profound read on model calibration and simulation (e.g., Sun and Sun, 2015).

It is clear that good and reliable ROI placement is a prerequisite when fitting the resulting TAC data to a model. Therefore, it is of great importance to know which part of the plant is being imaged inside the FOV. Some PET systems are combined with a CT or MRI module which facilitates this process because anatomical images can be obtained aside from the functional PET data. For plant-PET studies, however, simple photographs can generally serve as a good reference instead of CT or MRI data. The importance of good ROI-drawing practices is exemplified in Figure 7C which shows the branch segment that was imaged in Figure 7A. Without this image, it seemed obvious to start drawing ROIs from the point where the highest activity was measured (gray dotted ROIs). However, in these ROIs a petiole originates from the branch and due to the limited spatial resolution of the PET system (*∼* 1 – 3 mm), the tracer uptake inside the petiole and branch could not be resolved, resulting in TACs with a higher tracer uptake for these ROIs (gray TACs in Figure 7B). This would inevitably prompt incorrect parameter values upon model calibration. Therefore, it is advisable to select a branch segment without ramifications for ROI analysis.

Aside from studying *in vivo* dynamics of xylem-transported ^11^CO_2_ (Mincke et al., 2020), compartmental modeling has been applied in plant-PET studies to investigate the vulnerability of phloem characteristics, including the phloem speed to drought (Hubeau et al., 2018) and girdling (De Schepper et al., 2013a), tracer kinetics of plant carbon allocation, including carbon storage and export rate (Fares et al., 1988), and axial and lateral exchanges in transport pathways of plants (e.g., phloem) (Bühler et al., 2011, 2014).

## Conclusion

Positron emission tomography imaging is one of the key diagnostic tools used clinically to follow-up and treat diseases by making use of positron-emitting radioisotopes. The *in vivo* nature of this technique in combination with the ability to monitor dynamic processes has led to its application in plant science. Specifically, this imaging technique has already successfully shown its applicability to investigate the dynamic transport of nutrients, phytohormones as well as photoassimilates. However, in contrast to the numerous studies using laboratory animals and humans, the number of studies on plants is still limited. Therefore, the aim of this manuscript is to provide general insights on the opportunities of PET imaging as a tool for plant experiments and to guide the reader to start PET experiments on plants. To fully grasp PET imaging along with its potential and limitations, it is advised to have a profound read on the principles of PET or to follow a course on PET or biomedical imaging in general. Besides explaining the basics of PET imaging, this guide starts from planning the experiment, elucidates the different steps to execute plant-PET scans and completes with the quantification of the obtained data by means of mathematical frameworks. In this way, physiological parameters can be obtained that can otherwise not be measured *in vivo*, indicating the potential of plant-PET. We believe that *in vivo* imaging in combination with modeling, both at cell and organ scale, are necessary to advance our mechanistic understanding of plant physiology, including dynamics of xylem-transported CO_2_ and its relation to woody tissue photosynthesis, phloem characteristics as well as the effects of nutrients, hormones and both micro and macro environmental changes.

## Data Availability Statement

The raw data supporting the conclusions of this article will be made available by the authors, without undue reservation.

## Author Contributions

JM and KS designed the research and analyzed and interpreted the data. JC produced and administered the radioactivity. JM performed the experiments and wrote the first draft of the manuscript. All authors read and edited the manuscript before publication under supervision of KS.

## Conflict of Interest

The authors declare that the research was conducted in the absence of any commercial or financial relationships that could be construed as a potential conflict of interest.

## Footnotes

1 https://horosproject.org
