# Supplementary material for: Guide to Plant-PET Imaging Using 11CO2
Source: Front Plant Sci. 2021 Jun 2;12:602550. doi: 10.3389/fpls.2021.602550 (PMC8206809; doi:10.3389/fpls.2021.602550)
Supplement: Supplementary file 1 [file Data_Sheet_1.docx]

**Supplementary file 1: Radiation exposure**

Radiation exposure *X* at distance *d* [cm] from a source with activity *A* [mCi] is given by Eq. (S1) and is expressed in roentgen per hour (R h^-1^).

|  | $\text{X = }\frac{\text{A × Γ}}{\text{d}^{\text{ }\text{2}}}$ | (S1) |
| --- | --- | --- |

Where ﻿*Γ* is the exposure rate constant of the radionuclide (i.e. 7.18 ﻿R cm^2^ mCi^-1^ h^-1^ for ^11^C, ^13^N and ^15^O and 6.96 R cm^2^ mCi^-1^ h^-1^ for ^18^F, Saha, 2016). Cumulative exposure [R] over a period of time can be obtained by calculating the cumulative activity [mCi h] during that period which boils down to the integration of the universal law of radioactive decay Eq. (S2) from the start to the end of the exposure.

|  | $\text{A}_{\text{t}}\text{ = }\text{A}_{\text{0}}\text{ }\text{e}^{\text{-λt}}$ | (S2) |
| --- | --- | --- |

Where *A_t_* [mCi] is the remaining activity at time *t* [h] when starting activity was *A_0_* [mCi] of radionuclide with decay constant *λ* [h^-1^]. The latter can be derived from the half-life *t_1/2_* [h] = ln(2) / *λ*.

The cumulative exposure can be converted to absorbed dose (expressed in Gray - Gy) using experimentally determined conversion factors which are depending on the photon energy. For photon energies between 100 keV and 3 MeV the conversion factor remains more or less equal ~ 9.6 mGy R^-1^ (0.96 rad R^-1^ - Carron 2006). Since positron-emitting isotopes produce photon pairs of 511 keV this conversion factor can be used for PET isotopes.

Eventually, the effective dose can be obtained by multiplying the absorbed dose by a radiation weighting factor *W_R_* (i.e. 1 for photons) and a tissue weighting factor *W_T_* (i.e. 1 assuming that all body parts are uniformly irradiated).
