# Supplementary material for: Guide to Plant-PET Imaging Using 11CO2
Source: Front Plant Sci. 2021 Jun 2;12:602550. doi: 10.3389/fpls.2021.602550 (PMC8206809; doi:10.3389/fpls.2021.602550)
Supplement: Supplementary file 2 [file Data_Sheet_2.docx]

**Supplementary file 2: compartmental modelling**

The compartmental model shown in **Figure 8** is used to describe ^11^CO_2_-tracer movement in the four consecutive regions of interest (ROIs 1-4) within a branch segment shown in **Figure 7A**. Each ROI is divided in two compartments and the tracer concentration (*T_C_* in MBq) of each compartment is described by Eqs. (S3-4) where superscript denotes compartment number and subscript *i* the ROI number. The model is described by two parameters, i.e. xylem CO_2_ transport speed $\text{v}_{\text{CO2}}^{\text{ }}$ (mm min^−1^) and exchange parameter *a* (min^−1^). Through sap flow, ^11^CO_2_ enters and moves within the xylem conduits (i.e. compartment 1) of each ROI with transport speed $\text{v}_{\text{CO2}}^{\text{ }}$. Within each ROI ^11^CO_2_ can move from the xylem to surrounding chloroplast containing cells (i.e. compartment 2) through *a* where it is assimilated by woody tissue photosynthesis and stored.

|  | $\frac{\text{d}{\text{T}_{\text{C}}}_{\text{i}}^{\text{1}}}{\text{dt}}\text{=}\text{ }\frac{\text{v}_{\text{CO}\text{2}}^{\text{ }}}{\text{l}}\text{∙}{\text{T}_{\text{C}}}_{\text{i-1}}^{\text{1}} \text{-}\text{ }\frac{\text{v}_{\text{CO}\text{2}}^{\text{ }}}{\text{l}}\text{∙}{\text{T}_{\text{C}}}_{\text{i}}^{\text{1}} \text{-}\text{ }\text{a∙}{\text{T}_{\text{C}}}_{\text{i}}^{\text{1}}$ | (S3) |
| --- | --- | --- |
|  | $\frac{\text{d}{\text{T}_{\text{C}}}_{\text{i}}^{\text{2}}}{\text{dt}}\text{ }\text{=}\text{ }\text{a∙}{\text{T}_{\text{C}}}_{\text{i}}^{\text{1}}$ | (S4) |

Constant *l* is the axial length of the ROI (i.e. 4 mm). Parameter *a* represents the fraction of xylem-transported CO_2_ flowing across compartments and thus range from 0 to 1. Note that *a* is the net result of ^11^C-tracer flowing forth (*a_12_*) and back (*a_21_*) between both compartments because separate parameters (in- and outflow) are not identifiable. For the first timeframe, it is safe to assume that all measured tracer is present in compartment 1. This assumption was tested, and it was found that the parameter outcome was insensitive to whether initial activity was allocated to compartment 1 only or distributed across all three compartments. The tissue under study on which ROI analysis is executed (i.e. the branch in this example) should be checked to ensure that its dimensions do not change with ROI since all model parameters are assumed to be constant for each ROI as well as over the entire scan time. For each of the ROIs, parameters are assumed equal (i.e. steady state). When the process under study is not in steady-state a moving window (or box car) calibration may be performed. This method allows model calibration for parts of the total data set, i.e. calibration windows. For each calibration window a separate set of model parameter estimates is obtained which best described the data of the calibration window. In this way, insights can be obtained about the dynamic behaviour of the parameters. Note that this model allows CO_2_ gas-liquid interconversion (Hari *et al.* 1991; Levy *et al.* 1999) but does not differentiate between phases. Calibrated parameters should always be taken with caution given that other fluxes not accounted for by the model might bias parameter calibration.

ROI 1 is most proximally located with respect to the other ROIs and does not receive tracer from any other ROI. Because ^11^C-tracer is actually transported into this ROI, its total tracer amount (i.e. $T_{C_{total}}$) can be used to calculate the tracer concentration in the first compartment for each time step according to Eq. (S5). The change in tracer concentrations for the second compartment can be calculated according to Eq. (S4).

|  | ${T_{C}}_{1}^{1}={T_{C}}_{total}-{T_{C}}_{1}^{2}$ | (S5) |
| --- | --- | --- |
